# Complex Psychotropic Polypharmacy in Soweto-based community psychiatry clinics

**DOI:** 10.4102/sajpsychiatry.v31i0.2424

**Published:** 2025-07-18

**Authors:** Lee-Ann Mabulwana, Kagisho Maaroganye

**Affiliations:** 1 Department of Psychiatry, Faculty of Health Sciences, University of the Witwatersrand, Johannesburg, South Africa

**Keywords:** Complex Psychotropic Polypharmacy, prevalence rate of psychotropic polypharmacy, factors associated with psychotropic polypharmacy, community mental healthcare clinics, South Africa, psychotropic prescribing patterns

## Abstract

**Background:**

Psychotropic polypharmacy is increasing globally. In South Africa (SA), Complex Psychotropic Polypharmacy (CPP) prevalence is 36.3%. Being on CPP is associated with adverse drug reactions and worsened patient outcomes, but there exists limited knowledge on CPP risk factors in SA.

**Aim:**

To determine CPP prevalence and its associated clinical and sociodemographic factors in a community setting in SA.

**Setting:**

The study was conducted in five randomly selected community mental health clinics in Soweto township from 01 January 2021 to 31 December 2022.

**Methods:**

A retrospective study of 348 adult patient records was conducted from January 2021 to December 2022. Data on prescriptions, clinical and sociodemographic variables were extracted. Being on CPP was defined as having 3 or more psychotropics. Chi-square tests and logistic regression were used to identify factors associated with CPP.

**Results:**

The CPP prevalence was 25.3%. The most common CPP combination (26.1%) was oral antipsychotic, long-acting injectable antipsychotic and mood stabiliser prescriptions. Psychiatric diagnosis was significantly associated with CPP, *p*-value = 0.012. The most common adverse drug effect was associated with use of anticholinergic drugs (13.2%) of which 28.3% were on CPP. Those who were widowed or divorced were 4.3 times more likely to be on CPP compared to those never married (single) Adjusted odds ratio (AOR) = 4.3 (95% CI:1.2–16.1).

**Conclusion:**

There is notably high prevalence of CPP. The risk of adverse effects rises with an increase in the number of medications. Evidence-based prescribing and periodic review of medication should be emphasised.

**Contribution:**

Increased monitoring of prescribing practices is needed to determine if the practice of Complex psychotropic polypharmacy (CPP) prescription is in line with treatment guidelines for psychiatric diagnosis.

## Introduction

The increase of psychotropic polypharmacy globally, especially in high-income countries, remains a concern. Psychotropic polypharmacy has been variously defined as the concurrent administration of two or more psychiatric medications in a single patient^1^ whilst complex psychotropic polypharmacy (CPP) as the concomitant use of either three or more, or four or more psychotropic medications.^2,3,4,5,6^ The use of three or more psychotropic medications per patient has been the most used CPP definition.^7,8,9^

Studies from high-income countries have reported the growing trend of psychotropic polypharmacy. Multiple studies globally have reported that the prevalence of psychotropic polypharmacy ranges from 46.9% to 93.8%.^4,7,8,9,10,11^ Being on CPP (three or more psychotropics) drugs has increased from 34.9% in 1981–1990 to 49.7% in 1991–2000, while psychotropic monotherapy steadily decreased from 31.1% to 19.6% during the same period.^12^ In the United States, an office-based psychiatric practices study found a significant increase in the proportion of patients prescribed Complex psychotropic polypharmacy (CPP) (three or more psychotropics) from 16.9% in 1996–1997 to 33.2% in 2005–2006.^13^

Ayenew et al. found the prevalence rates of antipsychotic polypharmacy (APP) to be 40.6% among patients with schizophrenia in Africa.^14^ A review study by Fornaro et al. on psychotropic polypharmacy in mostly hospitalised bipolar patients reported that 22% of patients were on three or more psychotropic medications and 18% were on four or more psychotropic medications.^4^ Adeponle et al. found psychotropic polypharmacy to be as high as 92%, where 30.2% of patients were on three medications, 28.1% were on four medications, 6.1% were on four or more medications and 0.3% were on six medications in Nigeria.^10^

A retrospective record review study conducted at a psychiatry tertiary hospital in Johannesburg found psychotropic polypharmacy (>2 drugs) to be very high at 93.8% and CPP (four or more psychotropics) to be 36.37%.^7^ Veyej and Moosa^15^ examined specifically antipsychotic prescribing patterns in community clinics in Johannesburg, South Africa (SA). They found a very high prevalence of psychotropic polypharmacy (long-acting injectable antipsychotic and another psychotropic medication) at 90.3%, while the APP prevalence rate was 53.9%.^15^ Two retrospective studies conducted in Western Cape province, SA, found the prevalence of APP (two or more antipsychotics) to remain similar, ranging from 28.4% to 28.6% over a period of 8 years.^16,17^

Although polypharmacy may be necessary for refractory illness, when treating various symptoms or when treating co-occurring mental illnesses,^18^ still there may be adverse outcomes. Polypharmacy may have a detrimental impact on medication adherence and may raise the risk of adverse drug reactions, including cardiac concerns like arrhythmias or Q wave and T wave interval prolongation on electrocardiogram.^18,19^ Among the elderly, polypharmacy is associated with cognitive decline and diminished mobility.^20^ Side effects can make adult patients less likely to follow their treatment plans, which may lead to poor treatment outcomes.^21^

Several studies have identified different factors associated with being on CPP. Mojtabai et al.^13^ and Kim et al.^2^ have identified that being on CPP (three or more psychotropics) is associated with older age (> 50 years), is predominant among women because of their increased health-seeking behaviours^7^ and is more common in individuals with psychiatric comorbidities.^2,13^ A South African study by Armstrong et al. found that APP was more common in younger male patients diagnosed with schizophrenia.^17^

This study aims to determine the prevalence of CPP (defined as the use of three or more psychotropic medications), identify associated adverse outcomes and determine risk factors (including specific disorders) associated with being on CPP in a South African community health district. The findings may inform further research into contributing factors and support clinician training where prescribing patterns deviate from established treatment guidelines.

## Research methods and design

### Objectives

- To describe the sociodemographic, clinical characteristics and the prescribed medications of the study sample.
- To determine the prevalence rate of complex (the use of three or more psychotropic medications) psychotropic polypharmacy.
- To determine the sociodemographic and clinical factors associated with Complex Psychotropic Polypharmacy.

### Study design, setting and population

A retrospective study of patients’ clinical records was conducted. The records retrieved were for patients aged 18 years and older. The study was conducted in the community mental health clinics within the Soweto township during the period from 01 January 2021 to 31 December 2022.

Soweto is the largest township in Johannesburg, South Africa. Within the Johannesburg Metro district, the Witwatersrand University’s Psychiatry department is affiliated with 25 community mental health clinics. Soweto community was a preferable study site for this research because of easy accessibility and high volume of patients. Community mental health services are provided at eight primary care centres in Soweto. Five of the eight clinics (Mofolo clinic, Zola clinic, Orlando clinic, Meadowlands clinic and Chiawelo clinic) were randomly selected and used as the sample for this investigation.

Black individuals from lower socioeconomic backgrounds make up the majority of the patients served by these clinics. These facilities serve as community-based psychiatric clinics with specialised mental health services. Community mental health services are designed to treat people who need specialised psychiatric care because of severe mental diseases or significant psychological suffering.^22^ The clinics provide community-based psychiatric care to patients discharged from tertiary hospitals, such as general and speciality psychiatric facilities and also take referrals from primary healthcare practitioners. Additionally, some patients who live in residential care facilities operated by non-profit organizations (NPOs) and have psychiatric conditions are also treated at these clinics. Psychiatric medical officers and registrars work under the usually remote supervision of psychiatrists, and they are the main prescribers of psychotropic medications.

### Study sampling strategy and sample size

The study used a convenience sampling method to select the participants. The researcher visited to each of the five clinics and retrieved data from hard copies of patients’ clinical files that had been recently reviewed by mental healthcare providers within the previous 2 months. The patients’ files were stored in the filing cabinets at the respective clinics.

Patient records for those aged 18 years and older, all psychiatric-related diagnoses and records from patients seen during the study period were included in the study. Records from patients who had clinic consultations for at least a year were included. All files of the patients who did not meet the inclusion criteria were excluded from this study.

Cochran’s formula was used to calculate the sample size (equation 1):


$$n=\frac{z^{2}*\hat{p}(1-\hat{p})}{\varepsilon^{2}}$$ [Eqn 1]

where: *p* is the expected proportion or prevalence assumed to be 28.7%, which is the average of (21%–36.3%) the CPP rate found in studies that researched CPP,^4,5,7^ *Z*-score is a value from the normal distribution related to the significant level set at 1.96 (two-tailed 95% confidence interval or alpha = 0.05), *E* is the margin of error (0.05). Therefore, *n* = (1.96)^2^ (0.2865) (0.7135)/(0.05)^2^ = 315 patients’ files will detect statistical significance at the 5% level at a low effect size.

The sample size of 315 was divided between the five identified mental health clinics in Soweto. To determine the minimum sample from each of the five mental health clinics in Soweto, we used a proportional representation of each clinic towards the total population size. Chiawelo clinic needed at least 136 patient files, Mofolo clinic needed at least 78 patient files, Zola clinic needed at least 43 patient files, Orlando clinic needed at least 41 patient files and Meadowlands clinic needed at least 17 patient files.

### Data collection

Data were retrieved from clinical files of outpatients with mental illnesses. The data were manually extracted from patient files and included information on primary psychiatric diagnoses, sociodemographic details, such as age, gender and socioeconomic status, comorbid medical and psychiatric conditions, the number and types of psychotropic medications prescribed and any documented side effects of these medications. The diagnosis documented was the working diagnosis made from the clinical assessment by the treating doctor.

For the purposes of this study, we will regard Promethazine as a psychotropic as it was mostly used as a sedative at the different clinics where data were collected.

The data were captured directly into a Microsoft Excel spreadsheet for further analysis.

### Data analysis

The CPP prevalence was calculated as a percentage of the total sample, with 95% confidence interval (CI). Categorical data were summarised using frequencies and percentages. Factors associated with being on CPP were assessed using Pearson’s chi-square test for independence and logistic regression. Three multiple logistic regression models were fitted to identify factors that can predict being on CPP. Data analysis was performed using STATA version 18. The statistical significance was set at 0.05.

### Ethical considerations

Ethical clearance to conduct this study was obtained from the University of the Witwatersrand Human Research Ethics Committee (Medical) (No. M230843). The study also obtained permission to collect data from the clinical manager of the community-based mental health services in the Johannesburg District and the National Health Research Database (No. GP_202310_010). Participant’s personal information anonymity and confidentiality were achieved by allocating numbers to the different clinical records and the data collected solely by the principal investigator.

## Results

A total of 348 patients’ clinical records, from five different outpatient clinics, were retrieved: Chiawelo clinic accounted for 42.8% (*n* = 149), 23.3% (*n* = 81) from Mofolo clinic, 14.4% (*n* = 50) from Zola clinic, 13.5% (*n* = 47) from Orlando clinic and 6% (*n* = 21) from Meadowlands clinic.

### Complex Psychotropic Polypharmacy prevalence rate

**FIGURE 1:**
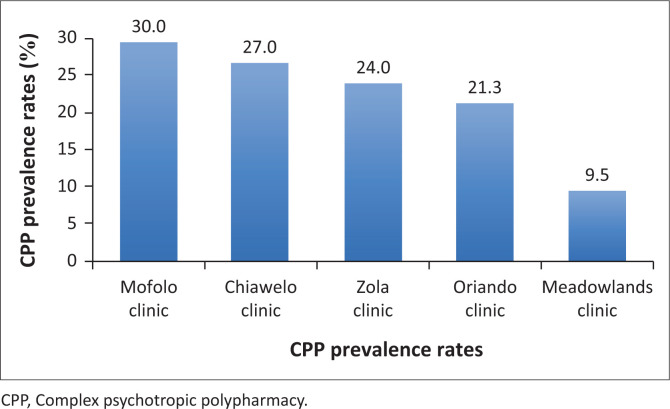
The CPP prevalence distribution at each clinic.

The CPP (three or more psychotropics) prevalence was found to be 25.3% (*n* = 88) with a 95% CI of 20.8% to 30.2%. Figure 1 shows the CPP prevalence distribution chart across the five clinics. Mofolo clinic had the highest CPP prevalence of 30%, while the Meadowlands clinic had the least CPP prevalence of 9.5%.

Overall, 29 different psychotropic combinations were identified. The CPP ranged from three to six psychotropics – 20.98% (*n* = 73) had three psychotropic prescriptions, while 3.74% (*n* = 13) had four psychotropic prescriptions. Table 1 shows the CPP distributions among the psychiatric patients. The most common CPP was a combination of oral antipsychotic, long-acting injectable antipsychotic and mood stabiliser, which was observed in 26.1% (*n* = 23) of the patients.

**TABLE 1:** Pattern of Complex Psychotropic Polypharmacy prescriptions.

Variable	Frequency (*n*)	Percentage (%)	CPP prescribed
Oral antipsychotic + Long-acting injectable antipsychotic + Mood stabiliser	23	26.1	3
Oral antipsychotic + Long-acting injectable antipsychotic + Antidepressant	7	8.0	3
Oral antipsychotic + Mood stabiliser + Antidepressant	7	8.0	3
Oral antipsychotic + Mood stabiliser + Promethazine	6	6.8	3
Oral antipsychotic + 2 Mood stabilisers	5	5.7	3
Oral antipsychotic + 2 Antidepressants	4	4.5	3
Long-acting injectable antipsychotic + Mood stabiliser + Promethazine	4	4.5	3
Oral antipsychotic + Antidepressant + Promethazine	3	3.4	3
Oral antipsychotic + Long-acting injectable antipsychotic + Benzodiazepine	3	3.4	3
Oral antipsychotic + Mood stabiliser + Benzodiazepine	3	3.4	3
Oral antipsychotic + Long-acting injectable antipsychotic + 2 Mood stabilisers	2	2.3	4
Oral antipsychotic + Long-acting injectable antipsychotic + Antidepressant + Promethazine	2	2.3	4
2 Oral antipsychotics + Mood stabiliser	2	2.3	3
Oral antipsychotic + Long-acting injectable antipsychotic + Promethazine	2	2.3	3
Oral antipsychotic + Long-acting injectable antipsychotic + 2 Mood stabilisers + Promethazine	1	1.1	5
Oral antipsychotic + Long-acting injectable antipsychotic + Mood stabiliser + Promethazine	1	1.1	4
Oral antipsychotic + Long-acting injectable antipsychotic + Mood stabiliser + Benzodiazepine	1	1.1	4
Oral antipsychotic + Long-acting injectable antipsychotic + Mood stabiliser + Antidepressant	1	1.1	4
2 Oral antipsychotics + Long-acting injectable antipsychotic + Mood stabiliser + Benzodiazepine + Promethazine	1	1.1	6
Oral antipsychotic + 2 Mood stabiliser + Promethazine	1	1.1	4
Antidepressant + Mood stabiliser + Benzodiazepine + Promethazine	1	1.1	4
Oral antipsychotic + Mood stabiliser + Antidepressant + Promethazine	1	1.1	4
Long-acting injectable antipsychotic + Mood stabiliser + Antidepressant + Promethazine	1	1.1	4
2 Antidepressants + Mood stabiliser	1	1.1	3
2 Oral antipsychotics + Long-acting injectable antipsychotic	1	1.1	3
2 Oral antipsychotics + Promethazine	1	1.1	3
Oral antipsychotic + Mood stabiliser + Promethazine	1	1.1	3
Oral antipsychotic + Antidepressant + Benzodiazepine	1	1.1	3
Long-acting injectable antipsychotic + Mood stabiliser + Antidepressant	1	1.1	3

CPP, Complex Psychotropic Polypharmacy.

### Sociodemographic, clinical and medication prescription patterns

The demographic and clinical characteristics of the patients is shown in Table 2, of the 348 patients, 58.6% (*n* = 204) were males. Most patients (26.2%, *n* = 91) were aged between 50–99 years, majority of them were single (72.7%, *n* = 253), most patients (37.1%, *n* = 129) had secondary education and most of them (40.8%, *n* = 142) were receiving disability grants.

**TABLE 2:** Demographic and clinical characteristics of the patients by Complex Psychotropic Polypharmacy prescription.

Variables	Total		No CPP		CPP		*χ* ^2^	*df*	*p*
	*n*	%	*n*	%	*n*	%			
**Gender**									
Female	137	39.4	98	37.7	39	44.3	1.052	1	0.305
Male	204	58.6	156	60.0	48	54.6	-	-	-
Not specified	7	2.0	6	3.3	1	1.1	-	-	-
**Age (years)**									
< 30	53	15.2	39	15.0	14	15.9	-	-	-
30–39	74	21.3	50	19.2	24	27.3	4.399	4	0.355
40–49	67	19.3	48	18.5	19	21.6	-	-	-
50–59	91	26.2	71	27.3	20	22.7	-	-	-
> 60	60	17.2	49	18.9	11	12.5	-	-	-
Not specified	3	0.9	3	1.2	-	-	-	-	-
**Marital status**									
Single	253	72.7	191	73.5	62	70.5	-	-	-
Married	35	10.1	24	9.2	11	12.5	2.989	3	0.393
Widowed or divorced	12	3.5	7	2.7	5	5.7	-	-	-
Not specified	48	13.8	38	14.6	10	11.4	-	-	-
**Education level**									
Primary	59	17.0	46	17.7	13	14.8	-	-	-
Special needs	10	2.9	7	2.7	3	4.4	1.302	4	0.861
Secondary	129	37.1	93	35.8	36	40.9	-	-	-
Tertiary	25	7.2	18	6.9	7	8.0	-	-	-
Not specified	125	35.9	96	36.9	29	33.0	-	-	-
**Income source**									
Disability grant	142	40.8	103	39.6	39	44.3	-	-	-
Employed	82	23.6	67	25.8	15	17.1	4.281	3	0.233
None	101	29.0	71	27.3	30	34.1	-	-	-
Not specified	23	6.6	19	7.3	4	4.6	-	-	-
**Psychiatry diagnoses**									
Psychotic disorder	183	52.6	146	58.0	37	42.1	-	-	-
Bipolar disorder	57	16.4	36	76.1	21	23.9	-	-	-
Depressive disorder	28	8.1	22	93.2	6	6.8	-	-	-
Anxiety disorder	7	2.0	4	96.6	3	3.4	16.275	6	**0.012**
Intellectual disability	36	9.3	22	84.1	14	15.9	-	-	-
Major neurocognitive	8	2.3	8	100.0	-	-	-	-	-
Personality disorder	22	6.3	13	89.8	9	10.2	-	-	-

Note: The figures in bold reflects the values with statistical significance.

CPP, Complex Psychotropic Polypharmacy.

Those on CPP, majority of them were males (*n* = 48, 54.6%), most of them were aged between 30–39 years (*n* = 24, 27.3%), majority were never married (*n* = 62, 70.5%), most had attained secondary education (*n* = 36, 40.9%) and 44.3% (*n* = 39) were receiving disability grants. None of the demographic characteristics were significantly associated with being on CPP at the bivariate level, *p*-values > 0.05.

**FIGURE 2:**
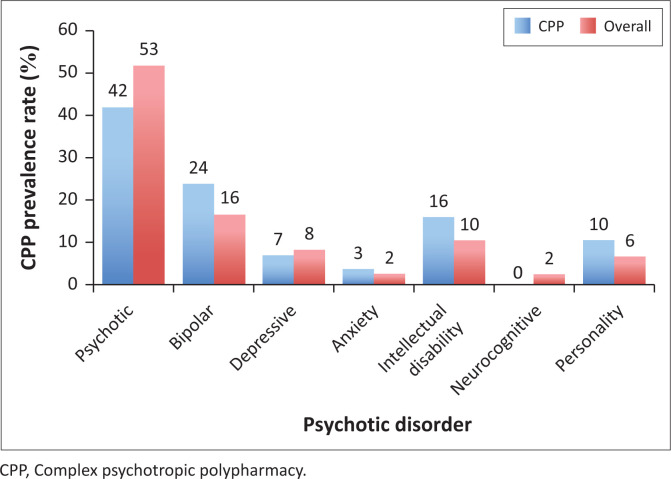
Percentage distribution of the different types of psychiatry diagnoses of the patients.

Figure 2 displays the psychiatry diagnosis distribution. Psychotic disorders were the most common diagnoses (53%, *n* = 183) followed by bipolar disorders accounting for 16% (*n* = 57) overall. Among those on CPP, a similar pattern was observed with a high percentage in the psychotic disorders (42%, *n* = 37) followed by bipolar disorders (24%, *n* = 21). There was a significant association between being on CPP and psychiatry diagnosis at the bivariate level, *p* = 0.012.

Table 3 displays the multiple logistic regression results on sociodemographic factors associated with being on CPP. Those who were once married (widowed or divorced) were 4.3 times more likely to be on CPP compared to those who never married (single) adjusted odds ratio (AOR) = 4.3 (95% CI: 1.2–16.1).

**TABLE 3:** Multiple logistic regression to predict Complex Psychotropic Polypharmacy among psychiatry patients in Soweto.

Variable	Categories	AOR	95% CI	*p*
**Age (years)**				
	< 30	1.07	0.4-2.5	0.880
	30–39	1.28	0.6-2.7	0.514
	40–49	1 (ref)	-	-
	50–59	0.64	0.3-1.4	0.251
	> 60	0.78	0.2-2.6	0.688
**Marital status**				
	Single	1 (ref)	-	-
	Married	1.85	0.8-4.3	0.150
	Widowed or divorced	4.30	1.2-16.1	**0.030**
**Level of education**				
	Primary	1 (ref)	-	-
	Special needs	1.33	0.3-6.6	0.729
	Secondary	1.32	0.6-3.0	0.508
	Tertiary	1.19	0.4-3.8	0.765
**Income**				
	None	1 (ref)	-	-
	Disability grant	1.05	0.6-2.0	0.889
	Employed	0.52	0.2-1.4	0.183

Note: The figures in bold reflects the values with statistical significance.

AOR, Adjusted odds ratio; CI, confidence interval.

### Prescribing patterns

Table 4 presents the prescription patterns in terms of specific medications and types. The most common oral antipsychotic was Risperidone prescribed to 31.1% (*n* = 136) of the patients of which 27.2% were on CPP. Clozapine was prescribed to 18.0% (*n* = 90) of the patients of which 5.6% were on CPP.

**TABLE 4:** Prescribed medications for the psychiatry patients.

Medication type	Specific medication prescribed	Total		CPP	
		*n*	%	*n*	%
Oral antipsychotics	Risperidone	136	31.1	37	27.2
	Quetiapine	34	7.8	12	35.3
	Olanzapine	48	11.0	12	25.0
	Haloperidol	34	7.8	6	17.6
	Clozapine	90	20.5	5	5.6
	Amisulpride	9	2.1	4	44.4
Long-acting injectable antipsychotics	Flupentixol decanoate	81	18.5	34	42.0
	Zuclopenthixol decanoate	19	4.3	7	36.8
Antiepileptics	Sodium valproate	126	28.8	44	34.9
	Lamotrigine	17	3.9	10	58.8
	Carbamazepine	8	1.8	5	62.5
	Lithium	16	3.7	10	62.5
Antidepressants	Citalopram	31	7.1	22	71.0
	Fluoxetine	21	4.8	8	38.1
	Amitriptyline	13	3.0	8	61.5
	Promethazine	41	9.4	27	65.9
	Benzodiazepines	16	3.7	8	50.0

CPP, Complex Psychotropic Polypharmacy.

Antidepressants demonstrated significant complex polypharmacy. Citalopram was prescribed to 6.2% (*n* = 31), of which 71.0% were on CPP, indicating frequent co-prescription for mood and anxiety disorders. Amitriptyline was prescribed to 2.6% (*n* = 13) of which 61.5% were on CPP. Adjunctive medications and sedatives also contributed notably to polypharmacy. Promethazine was prescribed to 8.2% of patients (*n* = 41), of which 65.9% were on CPP, suggesting frequent use for sedation or extrapyramidal symptom management. Benzodiazepines, although prescribed to only 3.2% (*n* = 16) of patients, 50.0% of them were on CPP, likely reflecting their role in managing acute agitation and sleep disturbances. The most common long-acting injectable antipsychotic was the Flupentixol decanoate prescribed among 18.5% (*n* = 81) patients and 42% (*n* = 34) of them were on CPP. The most common antiepileptic was the Sodium Valproate prescribed among 28.8% (*n* = 126) patients, and 34.9% (*n* = 44) of them were on CPP.

Table 5 presents the list of medication side effects reported. The most common adverse effect was the anticholinergic side effect (13.2%, *n* = 46), of which 28.3% (*n* = 13) were on CPP. Metabolic side effects were present among 4.3% (*n* = 15) of the patients, but not so common among those on CPP. Tardive dyskinesia was present among 3.5% (*n* = 12) of the patients of which 33.3% (*n* = 4) were on CPP. There was no association between adverse drug effects and being on CPP, *p* = 0.150 (χ^2^ = 8.124, *df* = 5).

**TABLE 5:** Adverse effects.

Variable	Total		CPP-relative to total	
	Count	%	Count	%
Anticholinergic medication	46	13.2	13	28.3
Metabolic side effects	15	4.3	1	6.7
Other (sexual dysfunction, sleep disturbances, acute dystonia)	3	0.9	1	33.3
Neuroleptic-induced parkinsonism	5	1.4	-	-
Tardive dyskinesia	12	3.5	4	33.3
Others	10	2.9	5	50.0

CPP, Complex Psychotropic Polypharmacy.

## Discussion

The aim of this study was to determine CPP prevalence and associated clinical and sociodemographic factors in a community setting in South Africa. This retrospective record review of the prevalence rate and associated factors of CPP of psychiatry community clinics in Soweto had more younger and middle-aged, single and unemployed patients. The study sample might not have been a true representative of the psychiatry population as convenient sampling was conducted and only those with files at these clinics were included in the study. However, the sample had a comparable sociodemographic characteristics distribution similar to the study by Veyej and Moosa who conducted a study in Soweto, psychiatric community clinics.^15^

The prevalence of CPP was 25.3%. Compared to other studies conducted in Africa, studies that reported CPP prevalence as low as 6.6% and as high as 67.9%, our finding falls within the range.^23,24,25^ Our prevalence estimate is comparable (not significantly different) to a study conducted in Nigeria, which reported a CPP (three or more psychotropics) prevalence of 30.2%.^10^ This study puts forward that CPP prevalence is high and remains a concern in South Africa. The high prevalence of psychotropic polypharmacy results in an increased risk of potential drug–drug interactions,^26^ which is a healthcare concern. Psychotropic polypharmacy also increases the financial burden on the healthcare systems, especially in lower- and middle-income countries like South Africa. In South Africa, mental health services are only allotted roughly 5% of the entire health budget, and the majority of funds are utilised for in-patient care.^27^ Reducing the CPP may lower treatment expenses and lessen its part in blundering the healthcare system with additional costs.^28^ Some of the psychotropic polypharmacy cost interventions to consider include deprescribing some medications^29^ and exploring adjunctive non-pharmacological strategies like psychotherapy,^30^ which have shown some benefits among psychiatric patients.

The most common CPP prescription was oral antipsychotic/long-acting injectable antipsychotic/mood stabiliser (26.1%). Having APP and/or same-class polypharmacy is generally discouraged in prescribing guidelines.^30^ In South Africa, APP prevalence varies from 27.8% to 53.9%, with the most prescribed antipsychotic combination being long-acting injectable first-generation antipsychotics with oral first-generation antipsychotics, corresponding with the data above.^15,16,17^ In this study, and likely in other African countries, the increased prevalence of APP may be attributed to cultural factors. Patients often seek treatment from traditional healers and faith-based practitioners before turning to Western medicine at a later stage of their illnesses.^31^ This delay in seeking medical treatment may contribute to patients presenting with refractory or more severe symptomatology, prompting clinicians to prescribe multiple antipsychotics to manage the more severe manifestations of mental illness. Other factors include lack of access to mental health facilities close to where patients live, lack of education about options for treating mental illnesses and stigma towards seeking help at recognised mental health facilities.^31,32^

The most common psychiatry diagnoses found in this study were psychotic disorders (42%), bipolar disorder (24%) and intellectual disability disorders (16%). Patients with psychotic disorders often have complex symptomatology and psychiatric comorbidities that might necessitate management with multiple psychotropics^9,10^ and, hence end up being on CPP. Patients with bipolar disorders are on CPP due to the multifaceted nature of the bipolar disorder illness and the potential limitations of monotherapy treatments, leading to the use of multiple complex medications. This is consistent with existing literature, individuals diagnosed with bipolar disorder have high likelihood of being on CPP.^11,25^ Patients with intellectual disorders have a high prevalence of co-occurring mental health conditions, including challenging behaviours such as aggression or self-injurious behaviour.^33,34^ As a result, these patients often receive high doses of multiple psychotropic agents.^33,34,35^ At times, there could be off-label prescribing in individuals with intellectual disability.^33,35^ Non-medical strategies like behavioural and therapeutic support for challenging behaviours may assist in using less psychotropics,^36,37^ and it has also been shown that these positive effects of these interventions can be long-lasting.^37^

The most common adverse drug effect was most likely to be neuroleptic-induced extrapyramidal side effects as evidenced by the anticholinergic medication prescriptions (13.2%), of which 28.3% were on CPP (anticholinergic medications are recommended for the management of extrapyramidal side effects^30^). However, there was no significant association between adverse drug effects and being on CPP in the bivariate analysis. The poor association of adverse drug effects and being on CPP could be attributed to poor documentation of these adverse drug effects or patients reporting the physical symptoms to other clinicians as opposed to psychiatric medical officers or registrars.

Those who were once married (widowed or divorced) were 4.3 times more likely to be on CPP compared to those who never married (single). Those who were divorced exhibited higher instances of CPP. Alharbi et al. found that those who are divorced are more likely to receive polypharmacy treatment.^11^ This can be attributed to the possibility that divorced individuals may have less social support.^38^ Divorced individuals may have experienced emotional and financial stressors during the legal proceedings of separation, leading to increased risk for mood disorders, anxiety and substance use.^39^

In this study, age was found not to be associated with being on CPP. Other studies that looked at psychotropic polypharmacy found that younger age has also been identified as a potential risk factor for psychotropic polypharmacy in various populations, particularly among individuals with bipolar disorder^11^ and psychotic disorders.^11,40^ Our study also found lower CPP in the elderly; this finding could be explained by the fact that the elderly people in this study might not have been on CPP because of the increased risk of medical comorbidities in this age group and/or anticipated adverse drug to drug interactions caused by the age-related changes in pharmacodynamics and pharmacokinetics in older age.^41^ Moreover, community clinics do not have medications like anticholinesterase inhibitors, which are cognitive enhancers. To manage psychotropic polypharmacy in the elderly, deprescribing, medication education and reviewing of psychiatric medications periodically were found to be beneficial.^42,43^

### Limitations

The data recorded for some of the patients regarding clinical information were incomplete and not available in some patients’ records. There was an inconsistency in the clinical information captured for each patient. Some patients’ records indicated that individuals were collecting medications from other outpatient clinics for chronic and presumably physical conditions; however, there was no documentation of these medications in the files. Furthermore, certain patients were prescribed anticholinergics, specifically Orphenadrine, without documentation, justifying the rationale for its use. It remains unclear whether this medication was prescribed to address the side effects of antipsychotics or as a pre-emptive measure.

Soweto is a community that faces a significant burden of substance use disorders among young people; however, substance use was not well explored in this study. Many patients with substance use disorders demonstrate poor adherence to treatment, which could account for their failure to meet the inclusion criteria of having more than 2 years of follow-up at the clinic. A greater representation of these patients, who tend to develop psychotic disorders and are young, could have resulted in a higher prevalence of being on CPP.

## Conclusion

In conclusion, a quarter of the sample, 25.3%, was prescribed three or more psychotropics as shown in this study. Certain medication prescriptions were more likely to be found in patients with CPP. Patient diagnosis was also associated with CPP, with higher percentages in psychotic disorders, bipolar disorder and intellectual disability. This study underscores the need for detailed clinical documentation, monitoring of prescribing practices and use of appropriate clinical guidelines to mitigate risks and optimise treatment.

### Implications and recommendations

This study demonstrated that 25% of the patients were on CPP regimens. The frequent use of APP and co-prescription of mood stabilisers raise concerns about potential adverse drug reactions and drug–drug interactions, both of which can possibly occur. The off-label use of certain drugs and incomplete adherence to the Standard Treatment Guidelines may have also contributed to the high prevalence of CPP. Further research into the risks associated with being on CPP in South African outpatient settings will be beneficial. It is also recommended that a prospective study of both community and hospital patients be conducted, focusing on specific psychiatric diagnoses associated with polypharmacy, where patients may be followed for a year or two.
